# Localization of bleeding sites in patients with hemoptysis based on their chest computed tomography findings: a retrospective cohort study

**DOI:** 10.1186/s12890-016-0322-1

**Published:** 2016-11-25

**Authors:** Hyun Ju Seon, Yun-Hyeon Kim, Yong-Soo Kwon

**Affiliations:** 1 Department of Radiology, Chonnam National University Medical School, 42 Jebongro, Donggu, Gwangju, 61469 South Korea; 2 Department of Internal Medicine, Chonnam National University Medical School, 42 Jebongro, Donggu, Gwangju, 61469 South Korea

**Keywords:** Massive hemoptysis, Ground glass attenuation, Fungus ball, Malignancy

## Abstract

**Background:**

The aim of this study was to propose a localization strategy for bleeding sites in hemoptysis patients using their chest computed tomography (CT) findings.

**Methods:**

Between January 2005 and July 2009, the chest CT findings of 161 hemoptysis patients were retrospectively reviewed. Following chest CT, the lobe with the most prominent ground glass attenuation (GGA) or specific lesions with the potential to cause pulmonary hemorrhage were analysed to develop a localization strategy for bleeding sites. Fibre optic bronchoscopy (FOB) findings of active bleeding were used as the standard reference for the bleeding sites.

**Results:**

The concordance rate between the most prominent GGA and FOB findings was higher than that between specific lesions and FOB findings (Kappa value [*k*] = 0.751 vs. 0.448, *p* < 0.001). Among the specific lesions, there were high concordance rates between lung cancer and FOB findings (3/3, 100%) and fungus balls and FOB findings (8/9, 89%). The agreement of localization of the bleeding site between FOB findings and the localization strategy based on chest CT findings including the most prominent GGA, lung cancer and fungus balls, showed almost perfect (*k* = 0.904).

**Conclusions:**

The localization of bleeding sites in hemoptysis patients could be determined by chest CT findings such as the most prominent GGA, malignancy and fungus ball.

## Background

Massive hemoptysis is one of the most dreaded conditions among all respiratory emergencies and can have a variety of underlying causes [1, 2]. Although medical and surgical treatments have been tried for hemoptysis, the mortality rate increases significantly to about 40%, when surgery is undertaken as an emergency procedure [3, 4]. Recently, bronchial arterial embolism (BAE) has been established as a fairly effective treatment of choice [5, 6]. Localization of the specific artery could help to perform successful BAE and surgery. Therefore, the rapid and exact identification of the site causing active bleeding is essential for the successful treatment of patients with hemoptysis. With technological advances in computed tomography (CT), chest CT or CT angiography (CTA) is well known as a comprehensive, non-invasive method for evaluating hemoptysis patients including for detecting the primary causes of hemoptysis, determining the site of active bleeding, and planning surgery or BAE [7–10]. Moreover, CT evaluation is essential when fibre optic bronchoscopy (FOB) evaluation is difficult, especially in emergencies or when FOB findings are indeterminate or negative [11–13]. However, localization of bleeding sites using chest CT is not always easy. Especially in patients with extensive bilateral disease such as multiple sites of bronchiectasis or equivalent findings, localization of bleeding sites is more challenging [14]. Furthermore, cases with extensive bilateral or multifocal bronchiectases are more common in tuberculosis-prevalent areas than in non-prevalent areas, as are cases with complicated fungus balls associated with pre-existing bronchiectasis.

Therefore, the aim of this study was to propose a localization strategy for bleeding sites based on chest CT in patients with hemoptysis.

## Methods

### Patients and study design

We reviewed the medical records of patients with hemoptysis from January 2005 to July 2009. One hundred sixty-one patients with hemoptysis who had undergone chest CT and FOB were enrolled in this study. The median time interval between chest CT and FOB was 3 days (Interquartile range = 2–5 days).

The definition of massive hemoptysis varies widely according to different reports and it ranges between 100 and 1000 mL of blood in a 24-h period [15]. It is estimated that more than 100 mL of hemoptysis which can cause abnormal gas exchange and/or airway obstruction or hemodynamic instability [15]. So, we defined massive hemoptysis as more than 100 mL in 24 h.

### CT scanning and image analysis

A 64-multidetector CT (MDCT) scanner (Sensation Cardiac 64; Siemens, Forchheim, Germany) and a 4-MDCT scanner (LightSpeed Qx/I; GE Medical System, Milwaukee, Wisconsin) were used to evaluate the thorax and the upper abdomen from the level of the supraclavicular area to the level of the upper pole of the right kidney. Ninety-one of the 161 patients underwent conventional enhanced CT and 71 patients underwent CTA. Pre-enhanced high-resolution CT (HRCT) preceded conventional enhanced CT or CTA. All the patients underwent craniocaudal scanning in the supine position and at end-inspiratory suspension during a single-breath hold. Two chest radiologists, who were blinded to the FOB findings and who had more than 10 years’ experience reading thoracic CT scans, analysed the CT images. Final decisions regarding the findings were determined in consensus. Bronchial or non-bronchial systemic arteries identified by CTA were not evaluated in this study.

**Fig. 1:**
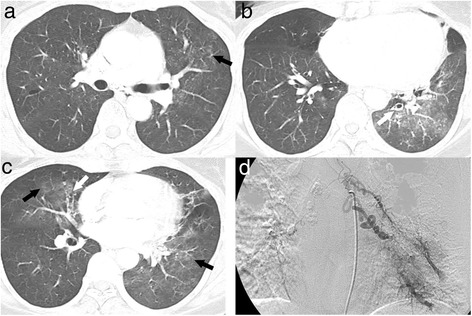
A 52-year-old woman with hemoptysis and multifocal bronchiectases. **a**–**c** Continuous chest computed tomography scans with a lung window setting show multifocal ground glass attenuations (GGAs, *black arrows* in **a**–**c**) in the lingular segment of the left upper lobe (LUL), the right middle lobe (RML), and the left lower lobe (LLL) with combined mild bronchiectasis in the RML (*white arrow* in **b**) and LLL (*white arrow* in **c**). The most prominent GGA site is the left lower lobe. **d** Selective left bronchial arteriogram shows tortuous and dilated left bronchial arteries with abnormal parenchymal staining of contrast media in the LLL

We evaluated abnormal radiological findings in the CT images as follows: First, we evaluated the presence or absence of ground glass attenuation (GGA), which is the result of the alveolar lumen filling with blood [7]. Among the lobes with GGA, we defined the site with the most prominent GGA as the lobe that has the largest extent or highest attenuation of GGA determined by the subjective opinion of each radiologist (Fig. 1). We presumed that the lobe with an active bleeding focus would have more prominent GGA than the other lobes. Discrepancies in the assessment were solved by consensus. We also checked for specific lesions that can cause hemoptysis such as bronchiectasis, active tuberculosis, old inflammatory lesions, fungus balls, malignancy and pneumonia. Bronchiectasis was defined as tubular or cystic bronchiectasis or bronchi twice as large as the diameter of the neighbouring arteries. Active tuberculosis was diagnosed when *Mycobacterium tuberculosis* was cultured from sputum or a FOB specimen with the typical radiological findings such as a tree-in-bud appearance [16]. A fungus ball was defined as a typical intracavitary round mass of soft-tissue density with an air-crescent sign visible on a chest CT scan [17]. The diagnosis of old inflammatory lesions caused by previous inflammation such as tuberculosis infections was based on a combination of the following findings: calcified granulomas, fibrocicatricial retractile linear opacities, bronchiectasis and pleural thickening [17]. Bronchiectasis with other sequelae of previous inflammation was classified as an old inflammatory lesion. Lung malignancy was designated as such only when pathological confirmation had been achieved. Pneumonia was defined according to the typical radiological finding and appropriate resolution with antibiotic therapy.

To locate the bleeding site, we compared concordance between the most prominent GGA and specific lesions. In the localization strategy for bleeding sites, the lesion which had a higher concordance with FOB findings was determined as a bleeding focus.

### Categorization of the FOB findings

Only direct visualization of active bleeding was regarded as a meaningful FOB finding that suggested the location of the exact bleeding focus. Indirect signs of bleeding such as hyperaemic mucosa or blood clot on FOB were also considered negative findings. Then, each result for predicting the bleeding focus by chest CT was compared with the FOB findings only in patients with visible active bleeding on FOB.

### Statistical analyses

Statistical analyses were performed with commercially available software (SPSS version 21.0; IBM Company, Chicago, Illinois). A bleeding focus on chest CT was decided by two criteria: the most prominent GGA site or the site with other abnormal findings that caused hemoptysis. FOB findings were used as a standard to determine the lobar localization of the active bleeding sites. For the evaluation of agreement between each of the FOB findings and each chest CT finding for the localization of the bleeding sites, the kappa value (Cohen’s kappa coefficient, k) was used. The level of agreement was assessed as follows: k < 0, no agreement; *k* = 0.0–0.20, slight agreement; *k* = 0.21–0.40, fair agreement; *k* = 0.41–0.60, moderate agreement; *k* = 0.61–0.80, substantial agreement; *k* = 0.81–1.00, almost perfect agreement. Statistical significance was set at *P* < 0.05.

## Results

### Baseline characteristics

The study included 94 men (58.4%) with a median age of 57.0 years (interquartile range [IQR] = 48.0–68.1 years). Among all the patients, 51 (31.7%) had massive hemoptysis (Table 1).

**Table 1:** Demographic characteristics and etiologies of patients with hemoptysis

Characteristics (*n* = 161)	Number or %
Age, median (IQR), years	57 (48.0–68.1)
Sex, male	94 (58.4%)
Massive hemoptysis	51 (31.7%)
Etiologies of hemoptysis (*n* = 125)	
Bronchiectasis	36 (28.8%)
Tuberculosis	31 (24.8%)
Old inflammatory lesions	30 (24.0%)
Pneumonia	11 (8.8%)
Fungus ball	9 (7.2%)
Malignancy	4 (3.2%)
Others^a^	4 (3.2%)

^a^paragonimiasis, catamenial hemoptysis, congenital cystic adenomatous malformation and broncho-esophageal fistula

### CT findings and the concordance between chest CT and FOB findings for the localization of bleeding sites

Of all 161 patients, GGA lesions were found in 118 (73.3%); specific lesions, in 125 (77.6%); and combined GGA and specific lesions, in 89 (55.3%). Seven patients (4.3%) did not have either GGA or specific lesions in their chest CT scans. Among patients with GGA lesions in their chest CT scan, 25 (21.2%) had GGA in a single lobe and 93 (78.8%) had GGA in multiple lobes. The median number of lobes with GGA per patient was 2 (IQR = 2–3). In 125 patients with specific lesions on their chest CT scan, the most common lesion was bronchiectasis (*n* = 36, 28.8%) followed by tuberculosis (*n* = 31, 24.8%), old inflammatory lesions (*n* = 30, 24.0%), pneumonia (*n* = 11, 8.8%), fungus ball (*n* = 9, 7.2%), malignancy (*n* = 4, 3.2%) and others (4, 3.2%) such as paragonimiasis, catamenial hemoptysis, congenital cystic adenomatous malformation and broncho-oesophageal fistula (Table 1).

**Fig. 2:**
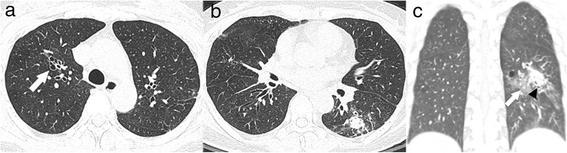
A 53-year-old woman with hemoptysis and a fungus ball. **a** Chest computed tomography scan showing mild bronchiectases in the right upper lobe (*arrow*) without evidence of haemorrhagic aspiration. **b** and **c** Axial chest computed tomography scan (**b**) and coronal reformation image (**c**) with a lung window setting showing an approximately 2.1 cm soft tissue nodule with the air-crescent sign (*arrow* head in **c**) and perilesional ground glass attenuations (*arrow*) in the superior segment of the left lower lobe (LLL)

**Fig. 3:**
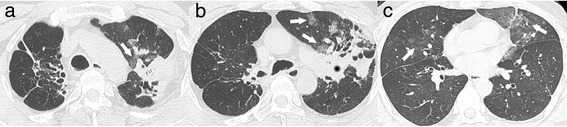
A 53-year-old man with hemoptysis and old inflammatory lesions. **a**–**c** Continuous high-resolution computed tomography scans showing old inflammatory lesions with traction bronchiectases and cicatricial volume loss in both the upper lobes and combined peribronchial consolidations (*star* in **b**) in the apicoposterior segment of the left upper lobe (LUL). Multifocal ground glass attenuations (GGAs, *arrows* in **a**–**c**) are seen in the LUL and right middle lobe (RML). The most prominent GGA site is the lingular segment of the LUL (*arrows* in **c**)

For the concordance between each lesion and FOB findings, the *k*-value was significantly higher in the most prominent GGA sites compared to that in the specific lesions sites (0.751 vs. 0.448, *p* < 0.001). Among 84 patients with specific lesions who had positive FOB findings for determining bleeding focus, malignancies or fungus balls (Fig. 2) had a high concordance with the FOB findings (3/3, 100% for malignancies and 8/9, 89% for fungus balls). However, old inflammatory lesions (Fig. 3), active tuberculosis, bronchiectasis (Fig. 1) and pneumonia revealed low concordance rates (47, 42, 41 and 33%).

### Diagnostic strategy for the localization of a bleeding site using chest CT

**Fig. 4:**
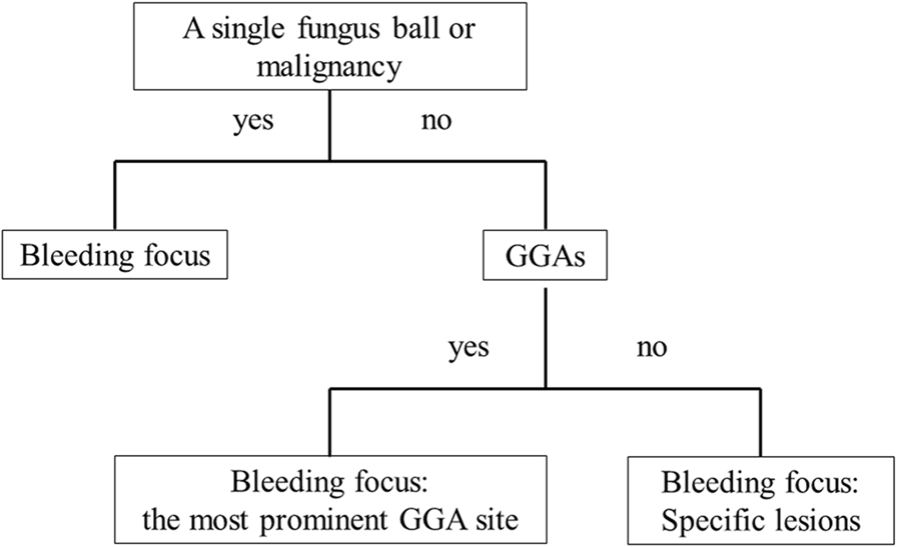
Localization strategy for bleeding sites in hemoptysis patients. In computed tomography findings, a single fungus ball or malignancy were the most probable sites for bleeding and in their absence or multiple lesions, the most prominent ground glass attenuation (GGA) was the next probable site. If GGAs did not exist, bleeding sites were determined using specific lesions other than a fungus ball or malignancy

Based on the primary results of our study, decisions were made to localise the bleeding focus using following criteria: (1) a single fungus ball or malignancy had priority over their multiple lesions, GGAs and other specific lesions, (2) if GGAs were present, the most prominent GGA was given priority over specific lesions excluding a single fungus ball or malignancy, and (3) in the absence of GGAs, the bleeding focus was determined by specific lesions excluding a fungus ball or malignancy (Fig. 4).

In 105 of the 107 patients with positive FOB findings, bleeding sites could be localised from chest CT using this diagnostic strategy. Of these 105 patients, 100 patients had concordance between the CT and FOB localization. The *k*-value for the localization of the bleeding focus between CT and FOB revealed almost perfect agreement (*k* = 0.904).

## Discussion

This is a large cohort study to suggest a rapid localization strategy using with CT finings in patients with hemoptysis. This study directly compared between CT and FOB findings in localization of bleeding sites, and active bleeding visualization by FOB were defined as standard reference of the bleeding sites. In this study, bleeding sites could be determined by the localization strategy using with CT finings such as the most prominent GGA, lung cancer and fungus ball.

Massive hemoptysis is associated with a high mortality rate, and accurate determination of the bleeding focus is important because many patients need emergency treatments such as surgical resection or BAE [3]. Various modalities are used for planning an emergency treatment in patients with massive hemoptysis, including chest radiography, chest CT, conventional angiography and FOB [9, 10]. They are complementary for the accurate determination of the bleeding sites and successful treatment to reduce the mortality. In addition to determining the bleeding focus, accurate analysis of the cause of the hemoptysis is important for effective care. Many studies revealed that chest CT could be a better tool for determining bleeding sites compared to other modalities in patients with hemoptysis [7, 12, 18–21]. Revel et al. [7] reported that the recognition rates of the bleeding sites by chest radiography, chest CT and FOB were 46, 70 and 73%, respectively, in patients with large or massive hemoptysis. However, all the patients in their study did not undergo all diagnostic modalities, and agreements among diagnostic modalities were not performed. Therefore, their study did not suggest a localization strategy for bleeding sites. Recently, MDCT revealed more accurate determination of bleeding sites in patients with hemoptysis due to precise delineation of bronchial and non-bronchial systemic arteries [22–24]. However, evaluation of bleeding sites in patients with bilateral lung abnormalities could be challenging because bilateral and multifocal bronchial arterial dilatations are also common [22–24]. Furthermore, the evaluation of each abnormal vessel including bronchial arteries is very time consuming and CTA by multidetector CT is often not available in emergencies [12]. Therefore, exact determination of the bleeding site is still difficult especially in patients with diseases causing bilateral lung abnormalities including tuberculosis and complications of tuberculosis such as bronchiectasis and fungus balls. Moreover, it is more challenging in cases with massive hemoptysis which can cause multiple haemorrhagic aspiration findings on chest CT. In our study, old inflammatory lesions, tuberculosis, bronchiectasis and pneumonia showed very low concordance rates (below 50%) with FOB findings due to multi-lobar involvements. However, the localization strategy in this study using findings of CT scan could accurately detect the bleeding sites, even in diseases presenting bilateral and multi-lobar involvements.

Localization of bleeding sites using chest CT findings, including GGA sites and specific lesions that could cause hemoptysis, has not been studied previously. According to our study, the most prominent GGA site could be an important clue for determination of the bleeding site, especially when there is no specific lesion which can cause bleeding, and there are multiple lesions such as multi-lobar involvements of bronchiectasis, tuberculosis and old inflammatory lesions. Importantly, sites with a malignancy or fungus ball coincided with the active bleeding sites on FOB in most cases. This result suggests that lesions with malignancy or fungus ball could be considered as an active bleeding focus.

Our study has some limitations. First, the bleeding sites for all patients could not be defined by FOB. Therefore, the agreement rates between FOB and chest CT findings could not be analysed. Since FOB could detect hemoptysis more effectively in patients with moderate to severe hemoptysis [2], our findings might have resulted from these patients. Second, the specific diagnosis was not confirmed by pathology in all cases. However, all cases of malignancies and fungus balls were pathologically confirmed. Third, this was a single centre retrospective study, which may limit the generalizability of our findings to other countries with a different disease prevalence.

## Conclusion

Bleeding sites in hemoptysis patients could be identified for emergency treatments including surgery and BAE based on the site of the most prominent GGA, malignancy or fungus ball on chest CT.

## Abbreviations

BAE: Bronchial arterial embolism
CT: Computed tomography
CTA: Computed tomography angiography
FOB: Fibre optic bronchoscopy
GGA: Ground glass attenuation
HRCT: High-resolution computed tomography
k: Kappa value
LLL: Left lower lobe
LUL: Left upper lobe
MDCT: Multidetector computed tomography
RML: Right middle lobe
